# Exploring causality with biliary atresia at different levels: two-sample Mendelian randomization study

**DOI:** 10.1136/wjps-2023-000754

**Published:** 2024-05-08

**Authors:** Shaowen Liu, Jiayinaxi Musha, Zhiru Wang, Xueting Wang, Tengfei Li, Jianghua Zhan

**Affiliations:** 1 Clinical School of Paediatrics, Tianjin Medical University, Tianjin, China; 2 Department of General Surgery, Tianjin Children's Hospital, Tianjin, China; 3 Urumqi City First People's Hospital (Urumqi Children's Hospital), Urumqi, China

**Keywords:** Genetics, Immunization, Information Technology, Pediatrics, Statistics

## Abstract

**Background:**

In recent years, Mendelian randomization (MR) has been widely used to infer causality of related disease risk exposures. However, this strategy has not been applied to biliary atresia (BA).

**Methods:**

Genome-wide association studies (GWAS) data of 41 inflammatory cytokines, 731 immune cell traits, and 1400 metabolites were obtained from public databases as exposure factors. The outcome information was obtained from a GWAS meta-analysis of 499 children with BA and 1928 normal controls. Inverse variance weighting was the primary causality analysis. Cochran Q-test, MR-Egger intercept, MR pleiotropy residual sum and outlier, and ‘leave-one-out’ analyses were used for sensitivity analysis. Reverse MR, MR-Steiger, and Linkage Disequilibrium Score were used to exclude the effects of reverse causality, genetic association, and linkage disequilibrium.

**Results:**

MR results showed that a total of seven traits had potential causal relationships with BA, including three inflammatory cytokines: eotaxin (odds ratio (OR)=1.45, 95% confidence interval (CI): 1.08 to 1.95, *p*_*FDR*_=0.18), G-CSF (OR=4.21, 95% CI: 1.75 to 10.13, *p*_*FDR*_=0.05) and MCP-1/MCAF (OR=1.53, 95% CI: 1.12 to 2.10, *p*_*FDR*_=0.14); three immune cell traits: CD8dim NKT/T cells ratio (OR=0.59, 95% CI: 0.45 to 0.77, *p*_*FDR*_=0.06), CD8dim NKT counts (OR=0.58, 95% CI: 0.43 to 0.78, *p*_*FDR*_=0.06), CD8dim NKT/lymphocyte ratio (OR=0.63, 95% CI: 0.49 to 0.81, *p*_*FDR*_=0.06); one metabolite: X-12261 levels (OR=2.86, 95% CI: 1.73 to 4.74, *p*_*FDR*_=0.06).

**Conclusions:**

In this study, eotaxin, G-CSF, MCP-1/MCAF, and X-12261 levels were shown to be risk factors for BA. However, CD8dim NKT/T cells ratio, CD8dim NKT counts, and CD8dim NKT/lymphocyte ratio were protective factors for BA. These findings provided a promising genetic basis for the etiology, diagnosis, and treatment of BA.

**WHAT IS ALREADY KNOWN ON THIS TOPIC**

- Biliary atresia (BA) exhibits inflammatory pathological features, and its occurrence may be closely associated with autoimmunity.
- Previous research confirmed the possibility of metabonomics as a non-invasive biomarker for the early detection of BA.
- Mendelian randomization (MR) analysis is a reliable causal assessment method, which has been widely used in various disease fields to find the possible causes of the disease. However, no study has reported the application of MR analysis in BA.

**WHAT THIS STUDY ADDS**

- The first MR analysis to explore causal relationships with BA from multiple levels.
- Eotaxin, G-CSF, MCP-1/MCAF, and X-12261 were risk factors for BA. CD8dim NKT/T cell ratio, CD8dim NKT count, and CD8dim NKT/lymphocyte ratio were protective factors for BA.

**HOW THIS STUDY MIGHT AFFECT RESEARCH, PRACTICE OR POLICY**

- This study conducted the MR analysis of BA from three different levels (inflammatory factors, immune cell traits, and metabolites), which would aid in understanding the pathogenesis of BA and provide a genetic basis for its diagnosis and treatment.

## Introduction

Biliary atresia (BA) is one of the most serious hepatobiliary diseases in neonates, which is characterized by progressive inflammatory fibrosis and occlusion of intrahepatic and extrahepatic bile ducts.^1^ The etiology of BA is still unclear, and the possible theoretical mechanisms include genetic variation, toxins, viral infection, autoimmune-mediated chronic inflammation or bile duct lesions, and abnormal bile duct development.^2^ Early diagnosis of BA can effectively improve the prognosis of children, and MMP-7 is the most promising early diagnostic index of BA; due to the lack of unified criteria, its clinical application is limited.^3 4^ In addition, there is currently no effective drug for the treatment of BA. Therefore, there is an urgent need to find the etiology of BA, new early diagnostic markers, and therapeutic targets.

In recent years, Mendelian randomization (MR) has been widely used to infer causality of related disease risk exposures. This process is done using publicly available genome-wide association studies (GWAS) datasets. Because genotypes are determined at conception, MR analyses are generally not susceptible to confounding factors.

In conclusion, BA exhibits inflammatory pathological features, and its occurrence may be closely associated with autoimmunity. In addition, previous research confirmed the possibility of metabonomics as a non-invasive biomarker for the early detection of BA.^5^ Therefore, this study conducted the MR analysis of BA from three different levels (inflammatory factors, immune cell traits, and metabolites), which would aid in understanding the pathogenesis of BA and provide a genetic basis for its diagnosis and treatment.

## Methods

### Research design

**Figure 1:**
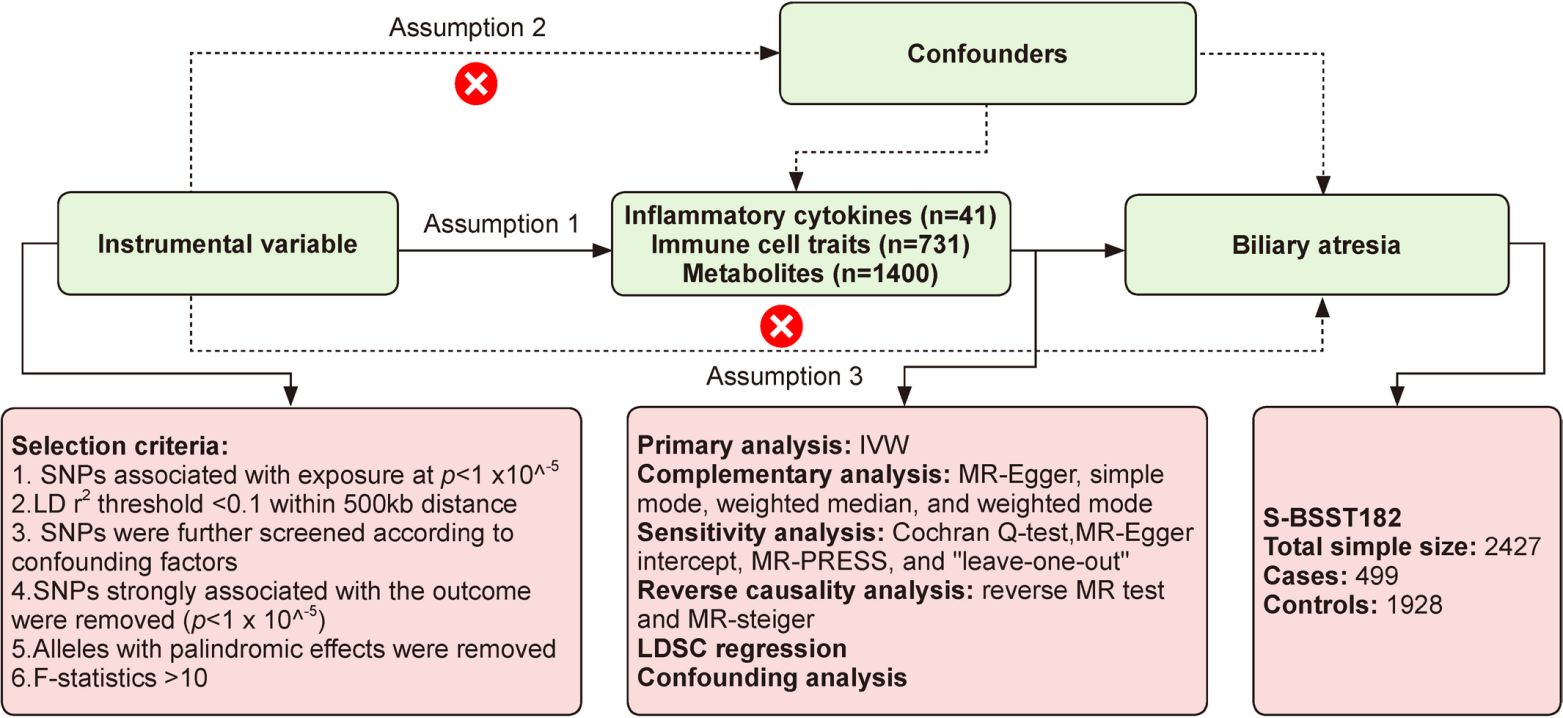
Flow chart of the Mendelian randomization (MR) study. IVW, inverse variance weighting; LD, linkage disequilibrium; LDSC, Linkage Disequilibrium Score; MR-PRESSO, MR pleiotropy residual sum and outlier; SNPs, single nucleotide polymorphisms.

Based on the two-sample MR analysis, we assessed 41 inflammatory cytokines, 731 immune cell traits, and 1400 metabolites for their potential causal relationships with BA as the outcome. The instrumental variables (IVs) used in MR analysis must adhere to three assumptions: (1) IVs must be exposure related, (2) IVs must be independent of other confounders, (3) IVs are unrelated to the outcome and can influence the outcome only through exposure.^6^ Figure 1 provides an overview of this MR study, and the study methods align with the Strengthening the Reporting of Observational Studies in Epidemiology-MR checklist.^7^

### GWAS data sources

We identified genetic predictors of 41 inflammatory cytokines from a comprehensive cytokine-related GWAS meta-analysis involving three independent cohorts. The study encompassed 8293 Finnish participants from the Cardiovascular Risk in Young Finns Study and the ‘FINRISK’ Studies (FINRISK1997 and FINRISK2002).^8^

Each immune trait from the GWAS data can be accessed publicly via the GWAS Catalog (https://www.ebi.ac.uk/gwas/, GCST0001391-GCST0002121).^9^ A total of 731 immune cell traits, comprising median fluorescence intensities reflecting surface antigen levels (n=389), relative cell (RC) counts (n=192), absolute cell (AC) counts (n=118), and morphological parameters (n=32), were included (online supplemental table 1). The GWAS for this immunologic profile used data from 3757 European individuals with no overlapping cohorts.

10.1136/wjps-2023-000754.supp1 Supplementary data

The GWAS dataset for 1400 metabolites was sourced from the study conducted by Chen *et al*.^10^ This represented the most comprehensive analysis of human metabolites, with complete summary statistics available through the GWAS Catalog (https://www.ebi.ac.uk/gwas/, GCST90199621-GCST90201020) in the public domain. The extensive GWAS involved 1091 metabolites and 309 metabolite ratios in 8299 individuals from the Canadian Longitudinal Study on Aging cohort. The dataset comprises 1091 metabolites, including 850 known metabolites and 241 unknown metabolites (online supplemental table 2). These known metabolites, categorized into peptide, nucleotide, amino acid, carbohydrates, cofactors and vitamin, energy, lipid, and xenobiotics metabolism, are based on the Kyoto Encyclopedia of Genes and Genomes database.

10.1136/wjps-2023-000754.supp2 Supplementary data

The GWAS dataset for BA was obtained from the study conducted by Chen *et al*.^11^ The first GWAS was conducted in a European-American cohort comprising 343 patients with isolated BA and 1716 controls. The second GWAS involved a European-American cohort with 156 patients presenting with BA and other extrahepatic abnormalities, alongside 212 controls. GWAS summary data can be accessed at https://www.ebi.ac.uk/biostudies/ (S-BSST182).

### Selection of IVs

The IVs selected for this MR analysis are based on three fundamental assumptions. Initially, single nucleotide polymorphisms (SNPs) with association thresholds at p<1×10^−5^ were extracted for each exposure. Subsequently, the clumping program within PLINK software (V.1.90) was employed to prune these SNPs, using a linkage disequilibrium (LD) r^2^ threshold of <0.1 within a 500 kb distance. The LD r^2^ was calculated based on the 1000 Genomes Project as a reference panel.^12^ Third, to validate the second hypothesis, we employed the ‘phenoscanner’ function to control for confounding factors. Fourth, to ensure the validity of the third hypothesis, we excluded SNPs strongly associated with the outcome variable (p <1×10^–5^). Additionally, alleles with palindromic effects were removed, and the explained variance (R^2^) and F-statistics parameters were used to assess the strength of IVs. A threshold of F-statistics >10 is generally recommended for MR analysis. Finally, we conducted MR analysis on metabolites comprising more than two SNPs, calculating R^2^ and F-statistics as follows:

$$R^{2}=\frac{2\times \beta^{2}\times EAF\times \left(1-EAF\right)}{\left[2\times \beta^{2}\times EAF\times \left(1-EAF\right)+2\times {\left(se\left(\beta \right)\right)}^{2}\times N\times EAF\times \left(1-EAF\right)\right]}$$

$$F=\frac{N-k-1}{k}\times \frac{R^{2}}{1-R^{2}}$$

Where β represents the effect size of the genetic variant of interest; EAF denotes the effect allele frequency of the genetic variant of interest; se (β) indicates the SE of the effect size of the genetic variant of interest; N stands for the exposure sample size; and k is the number of SNPs.

### MR analysis

This MR analysis primarily estimated the causal association using standard inverse variance weighting (IVW) methods.^13^ When the IVs satisfy all three assumptions of the valid IVs, the IVW approach can provide a consistent assessment of the causality of the exposure. However, if some IVs deviate from these assumptions, the analysis may yield inaccurate results. Hence, the following analyses were conducted: (1) heterogeneity among selected IVs was assessed using the Cochran Q-test with the corresponding p value. If the null hypothesis was rejected, a random-effect IVW was applied instead of a fixed-effect IVW.^14^ (2) Horizontal pleiotropy was evaluated through the intercept using the MR-Egger method to ensure that genetic variants were independently associated with both exposure and outcome.^15^ (3) Additional analyses using MR methods with different modeling assumptions and strengths (MR-Egger, simple mode, weighted median^16^, and weighted mode^17^) were performed to increase the stability and robustness of the results. (4) The MR pleiotropy residual sum and outlier (MR-PRESSO) method was used to identify and exclude horizontal pleiotropic outliers that might significantly influence the estimated results.^18^ (5) A ‘leave-one-out’ sensitivity analysis was employed to assess whether the results were influenced by any single SNP.^15^ Additionally, we conducted MR-Steiger and reverse MR tests to ascertain whether our results supported our hypothesis.

### Evaluation of genetic correlation and directionality

Although SNPs associated with BA were excluded in the selection of IVs, unrelated SNPs might have mediated the inheritance of BA. Therefore, to ensure that causal effects were not confounded by exposure consistency with the outcome, the SNP-based Linkage Disequilibrium Score (LDSC) was used to estimate coinheritance by Χ^2^ statistics for both traits.

### Confounding analysis

Although we evaluated horizontal pleiotropy in the MR results through various sensitivity analyses to detect SNPs violating the MR assumption, residual confounding SNPs might still exist. The ‘phenoscanner’ function was employed to assess whether each SNP was associated with known risk factors for BA, such as viral infection and maternal exposure to certain chemicals.^19^ If any SNP was found to be associated with the mentioned confounders (p<1×10^–5^), the MR analysis was re-executed after excluding these SNPs to validate the reliability of the results.

### Statistical analysis

Statistical analyses were conducted using R V.4.1.2 software (http://www.Rproject.org). MR analysis was carried out using the ‘TwoSampleMR’ package (V.0.5.7).^20^ IVW, weighted median-based, and model-based approaches were performed with the use of the ‘MendelianRandomization’ package (V.0.7.0).^21^ The false discovery rate (FDR)-adjusted p value, below 0.2, suggests statistical significance.^22^

## Results

### Causal assessment of 41 inflammatory cytokines for BA

**Figure 2:**
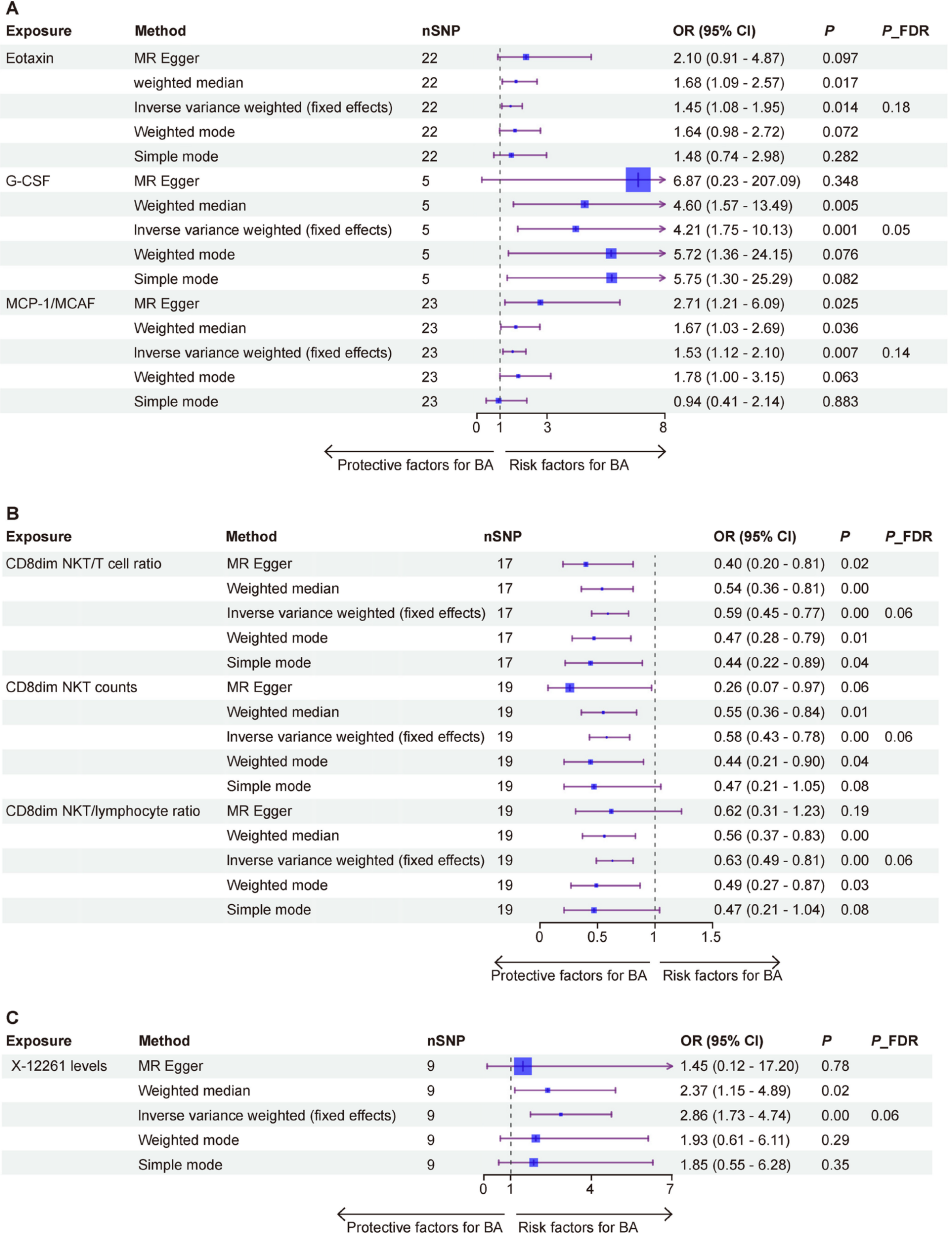
Forest plots of positive results from Mendelian randomization (MR) analysis. (A) Inflammatory cytokines, (B) immune cell traits and (C) metabolites. BA, biliary atresia; FDR, false discovery rate; SNP, single nucleotide polymorphism.

To investigate the causal relationship between inflammatory cytokines and BA, a two-sample MR analysis was conducted. Using IVW as the primary method, three suggestive associations were detected following FDR adjustment (*p*_*FDR*_<0.2); we identified three inflammatory cytokines as risk factors for the development of BA (figure 2A): eotaxin (odds ratio (OR)=1.45, 95% confidence interval (CI): 1.08 to 1.95, *p*_*FDR*_=0.18, *p*=0.014), G-CSF (OR =4.21, 95% CI: 1.75 to 10.13, *p*_*FDR*_=0.05, p=0.001) and MCP-1/MCAF (OR=1.53, 95% CI: 1.12 to 2.10, *p*_*FDR*_=0.14, *p*=0.007).

### Causal assessment of 731 immune cell traits for BA

To investigate the causal relationship between immune cell traits and BA, a two-sample MR analysis was employed. Using IVW as the primary analysis method, a total of 34 suggestive associations were identified, following FDR adjustment (*p*_*FDR*_<0.2); we identified three immune cell traits as risk factors for the development of BA, two of which were derived from RC counts and one from AC counts (figure 2B): CD8dim NKT/T cells ratio (OR =0.59, 95% CI: 0.45 to 0.77, *p*_*FDR*_=0.06, *p*=0.000), CD8dim NKT counts (OR =0.58, 95% CI: 0.43 to 0.78, *p*_*FDR*_=0.06, *p*=0.000), CD8dim NKT/lymphocyte ratio (OR=0.63, 95% CI: 0.49 to 0.81, *p*_*FDR*_=0.06, *p*=0.000).

### Causal assessment of 1400 metabolites for BA

To investigate the causal relationship between metabolites and BA, a two-sample MR analysis was conducted. Employing IVW as the primary analysis method, a total of 78 suggestive associations were identified following FDR adjustment (*p*_*FDR*_<0.2); we identified only one metabolite as a risk factor for BA development, and it came from the category of unknown metabolites (figure 2C): X-12261 levels (OR=2.86, 95% CI: 1.73 to 4.74, *p*_*FDR*_=0.06, *p*=0.000).

The detailed data of IVs were shown in online supplemental table 3. Generally, when statistical significance was observed in two other MR tests (p<0.05), the causal association is deemed robust. Notably, the association of MCP-1/MCAF, CD8dim NKT counts, CD8dim NKT/T cells ratio, CD8dim NKT/lymphocyte ratio, and X-12261 levels was deemed robust (online supplemental table 4). Although eotaxin and G-CSF were significant only in the IVW and weighted median methods (*p*<0.05), they still signified potential causal associations (online supplemental table 4).

10.1136/wjps-2023-000754.supp3 Supplementary data

10.1136/wjps-2023-000754.supp4 Supplementary data

### Heterogeneity test

Heterogeneity was tested using the Cochran Q-test. The p values of seven traits (eotaxin, G-CSF, MCP-1/MCAF, CD8dim NKT count, CD8dim NKT/T cell ratio, CD8dim NKT/lymphocyte ratio, and X-12261 level) were greater than 0.05, indicating that no significant heterogeneity was found (online supplemental table 4).

### Sensitivity analysis

**Figure 3:**
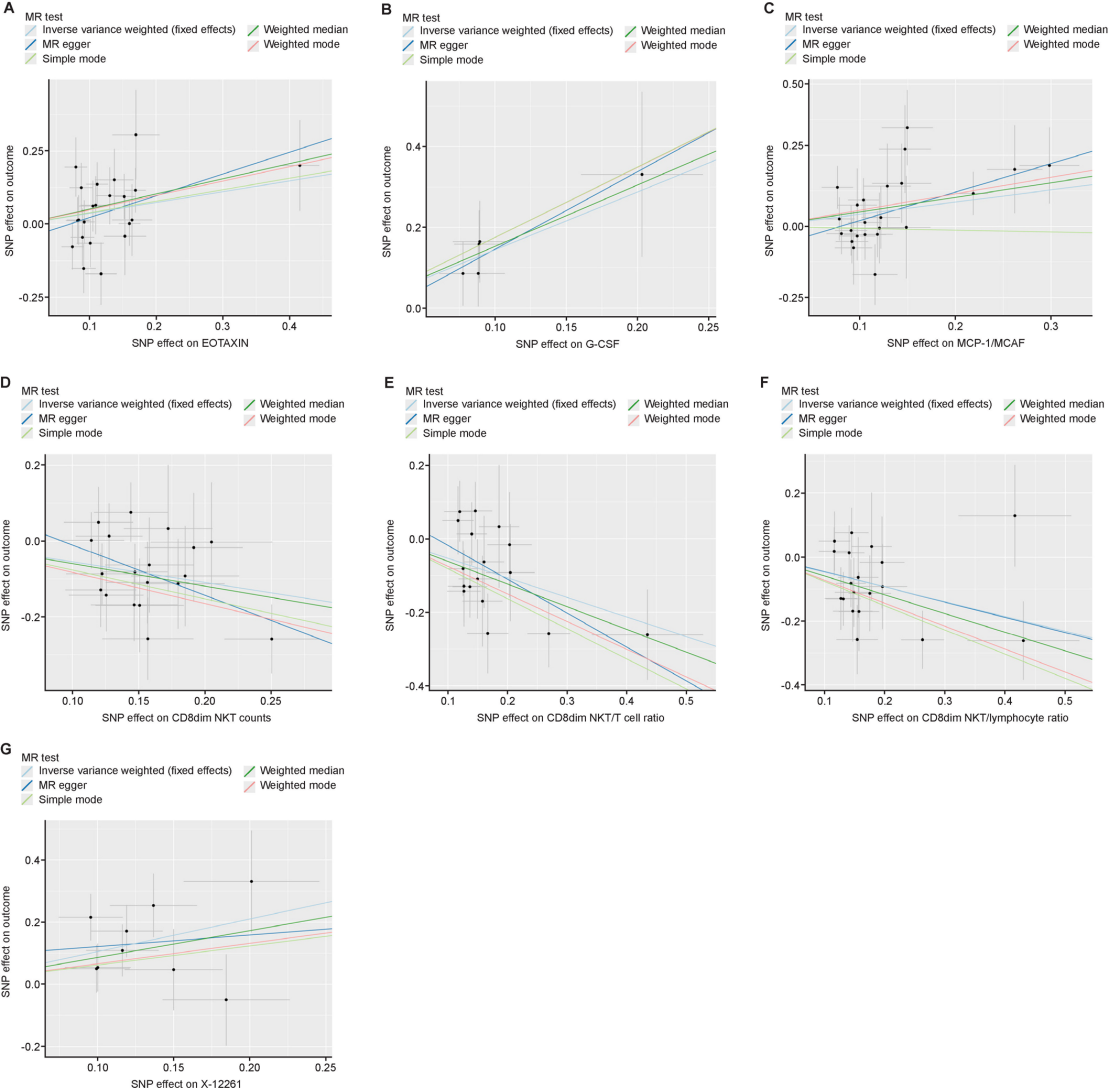
Scatter plots of positive results for Mendelian randomization (MR) analysis. (A) eotaxin, (B) G-CSF, (C) MCP-1/MCAF, (D) CD8dim NKT counts, (E) CD8dim NKT/T cells ratio, (F) CD8dim NKT/lymphocyte ratio and (G) X-12261. SNP, single nucleotide polymorphism.

Sensitivity analyses were performed to identify horizontal pleiotropy and potential outliers. All associations were scrutinized for potential horizontal pleiotropy using the MR-Egger intercept and MR-PRESSO global test. All p values in the results were greater than 0.05, indicating that there was no significant horizontal pleiotropy (online supplemental table 4). Additionally, we visualized scatter plots (figure 3), funnel plots (online supplemental figure 1), and ‘leave-one-out’ plot (online supplemental figure 2) from which no potential outliers and the possibility of horizontal pleiotropy were found.

10.1136/wjps-2023-000754.supp8 Supplementary data

10.1136/wjps-2023-000754.supp9 Supplementary data

10.1136/wjps-2023-000754.supp10 Supplementary data

### Reverse causality analysis

To test hypothesis 3 (‘IVs are unrelated to the outcome and can only influence the outcome through exposure’), MR-Steiger and reverse MR tests were conducted. Results indicated that in reverse causality analysis with BA as the exposure factor, the p values of the MR-Steiger for eotaxin, G-CSF, MCP-1/MCAF, and X-12261 levels were all below 0.05. And their p values of the reverse MR test were all above 0.05, confirming the study’s correct directionality (online supplemental tables 4 and 5). However, due to the lack of data for immune cell traits in the reverse MR test, their association with BA was verified solely through the MR-Steiger method. CD8dim NKT counts, CD8dim NKT/T cells ratio, and CD8dim NKT/lymphocyte ratio all had the p values of the MR-Steiger below 0.05, validating the study’s directionality (online supplemental table 4).

10.1136/wjps-2023-000754.supp5 Supplementary data

### Linkage Disequilibrium Score

The results of LDSC showed that eotaxin (R_g_=0.068, SE=0.110, p=0.537), G-CSF (R_g_=0.572, SE=0.313, p=0.068), MCP-1/MCAF (R_g_=−0.077, SE=0.081, p=0.342), and X-12261 levels (R_g_=−0.060, SE=0.161, p=0.711), which indicated that the MR estimates were not disturbed by the shared genetic component (table 1). We estimated the SNP–SNP heritability (proportion of variance attributed to genome-wide SNPs) for the four associations using LDSC. The SNP heritability of metabolites varied from 0.015 (G-CSF) to 0.167 (MCP-1/MCAF) (online supplemental table 6). The immune cell traits dataset was not analyzed as it lacked the necessary data for LDSC completion.

10.1136/wjps-2023-000754.supp6 Supplementary data

**Table 1:** Results of LDSC

Trait1	Trait2	R_g_	SE	P value
Eotaxin	BA	0.068	0.110	0.537
G-CSF	BA	0.572	0.313	0.068
MCP-1/MCAF	BA	−0.077	0.081	0.342
X-12261 levels	BA	0.060	0.161	0.711

BA, biliary atresia; LDSC, Linkage Disequilibrium Score.

### Confounding analysis

Although sensitivity analysis in this study was conducted to assess SNP estimates, to meet assumption 2 (‘IVs must be independent of other confounders’), we examined whether all SNPs associated with the seven traits were independent of BA risk factors (viral infection and maternal exposure to certain chemicals). Results indicated that none of the seven traits were associated with any confounding factors (online supplemental table 7).

10.1136/wjps-2023-000754.supp7 Supplementary data

## Discussion

Based on extensive publicly available genetic data, we investigated the causal relationship between various factors, including 41 inflammatory cytokines, 731 immune cell traits, and 1400 metabolites, and the disease outcome of BA. To our knowledge, this is the first MR analysis to explore causal relationships with BA at multiple levels. Our study identified three inflammatory cytokines (eotaxin, G-CSF, MCP-1/MCAF), three immune cell traits (CD8dim NKT counts, CD8dim NKT/T cells ratio, CD8dim NKT/lymphocyte ratio), and one metabolite (X-12261 levels) as having a significant causal relationship with BA.

The expression of each of the three inflammatory factors was a risk factor for BA, respectively, consistent with previous studies. Udomsinprasert *et al* 23 found that eotaxin, G-CSF, and MCP-1 levels were higher in the blood of children with BA. Increased eotaxin levels were notably linked to adverse outcomes such as jaundice, fibrosis, and portal hypertension. Moreover, the study identified MCP-1 as a potentially sensitive and specific biomarker for BA, with significantly upregulated relative mRNA expression observed in BA livers.

MCP-1 may play an important role in the development of BA with progressive liver fibrosis. Glucocorticoid treatment can regulate the expression of MCP-1 in the liver of cholestatic rats and reduce the infiltration of inflammatory cells.^24 25^ Ramm *et al* 26 found that bile acid-induced upregulation of hepatocyte-derived MCP-1 expression led to the recruitment of hepatic stellate cells and was a critical early event in the development of liver fibrosis. Jafri *et al* 27 found that chemokines expressed by rhesus rotavirus-infected cholangiocytes, such as MCP-1, might trigger host inflammatory processes leading to bile duct obstruction.

In a phase 1 study exploring Kasai-G-CSF sequential therapy for BA, G-CSF safely mobilized hematopoietic stem cells in Kasai children, potentially enhancing short-term biliary drainage and mitigating cholangitis.^28^ Nonetheless, the efficacy outcomes of sequential adjuvant Kasai and G-CSF therapy in phase 2 have yet to be determined. Intriguingly, another study observed high expression of G-CSF in the liver tissues of children with BA.^23^ While our findings suggest that G-CSF is a risk factor for BA, discrepancies between MR analysis and validation results might occur, influenced by factors such as insufficient sample size and small effect size. Hence, for G-CSF, we will further validate its relationship with BA in subsequent studies.

Our study revealed a decrease in the risk of BA with increasing CD8dim NKT counts, CD8dim NKT/T cells ratio, and CD8dim NKT/lymphocyte ratio. Notably, the association between CD8dim NKT counts and BA had not been previously reported. Previous research on the connection between BA and CD8+ T cells showed that a higher degree of CD8+ T cell infiltration in the bile duct of children with BA was correlated with better liver function.^29^ In addition, Kotb *et al* 30 observed a greater mean CD4+/CD8+ ratio in children who died within eighteen months after the Kasai procedure than in other children with BA.

Our study has several strengths. First, the two-sample MR study design helps reduce bias present in observational association studies, stemming from residual confounding and reverse causality. Second, we systematically investigated the causal relationship between multiple levels of data (including inflammatory cytokines, immune cell traits, and metabolites) and BA. Third, we employed seven MR methods to assess the robustness of causal associations and effect directions, including IVW, MR-Egger, weighted median, weighted mode, simple mode, MR-PRESSO, and MR-Steiger.

However, there are limitations to consider. First, we did not further subdivide BA considering the classification of the original data. Second, the power of IVs relies heavily on the sample size of GWAS, necessitating more data to enhance accuracy. Third, while MR analysis is reliable for assessing causality, it cannot replace randomized controlled trials (RCTs). Therefore, inferred causal relationships may not align with those observed in RCTs and require further validation in future studies. Fourth, our study relied on the Euro-American cohort for GWAS data, limiting the generalizability of our findings to other ethnic groups.

In conclusion, our study used publicly available GWAS data and MR analysis to identify three inflammatory cytokines (eotaxin, G-CSF, MCP-1/MCAF) and one metabolite X-12261 as risk factors for BA with a significant causal relationship. Three immune cell characteristics (CD8dim NKT counts, CD8dim NKT/T cells ratio, CD8dim NKT/lymphocyte ratio) also had a significant causal relationship with BA, and they were protective factors for BA. These findings contributed to a new genetic understanding of BA’s etiology, diagnosis, and potential treatment strategies. In our subsequent work, we will evaluate the accuracy of three inflammatory cytokines in the early diagnosis of BA using clinical samples. Furthermore, we will use in vivo and in vitro experiments along with multi-omics techniques to further investigate the involvement of these three inflammatory cytokines and CD8dim NKT cells in the pathogenesis of BA, exploring their specific roles in disease progression.

## Data Availability

Data are available in a public, open access repository. Data sources are included in the Methods section of the article. Further information is available from the corresponding author.
