# Supplementary material for: Exploring causality with biliary atresia at different levels: two-sample Mendelian randomization study
Source: World J Pediatr Surg. 2024 May 8;7(2):e000754. doi: 10.1136/wjps-2023-000754 (PMC11086552; doi:10.1136/wjps-2023-000754)
Supplement: Supplementary data [file wjps-2023-000754supp008.pdf]

Supplementary Figure 1 Funnel plots of positive results for Mendelian randomization analysis. A Eotaxin. B G-CSF. C MCP-1/MCAF. D CD8dim NKT counts. E CD8dim NKT/T cells ratio. F CD8dim NKT/lymphocyte ratio.

Supplementary Figure 2 “leave-one-out” analyses of positive results for Mendelian randomization analysis. A Eotaxin. B G-CSF. C MCP-1/MCAF. D CD8dim NKT counts. E CD8dim NKT/T cells ratio. F CD8dim NKT/lymphocyte ratio.
