# Supplementary figures and images for: Exploring causality with biliary atresia at different levels: two-sample Mendelian randomization study
Source: World J Pediatr Surg. 2024 May 8;7(2):e000754. doi: 10.1136/wjps-2023-000754 (PMC11086552; doi:10.1136/wjps-2023-000754)

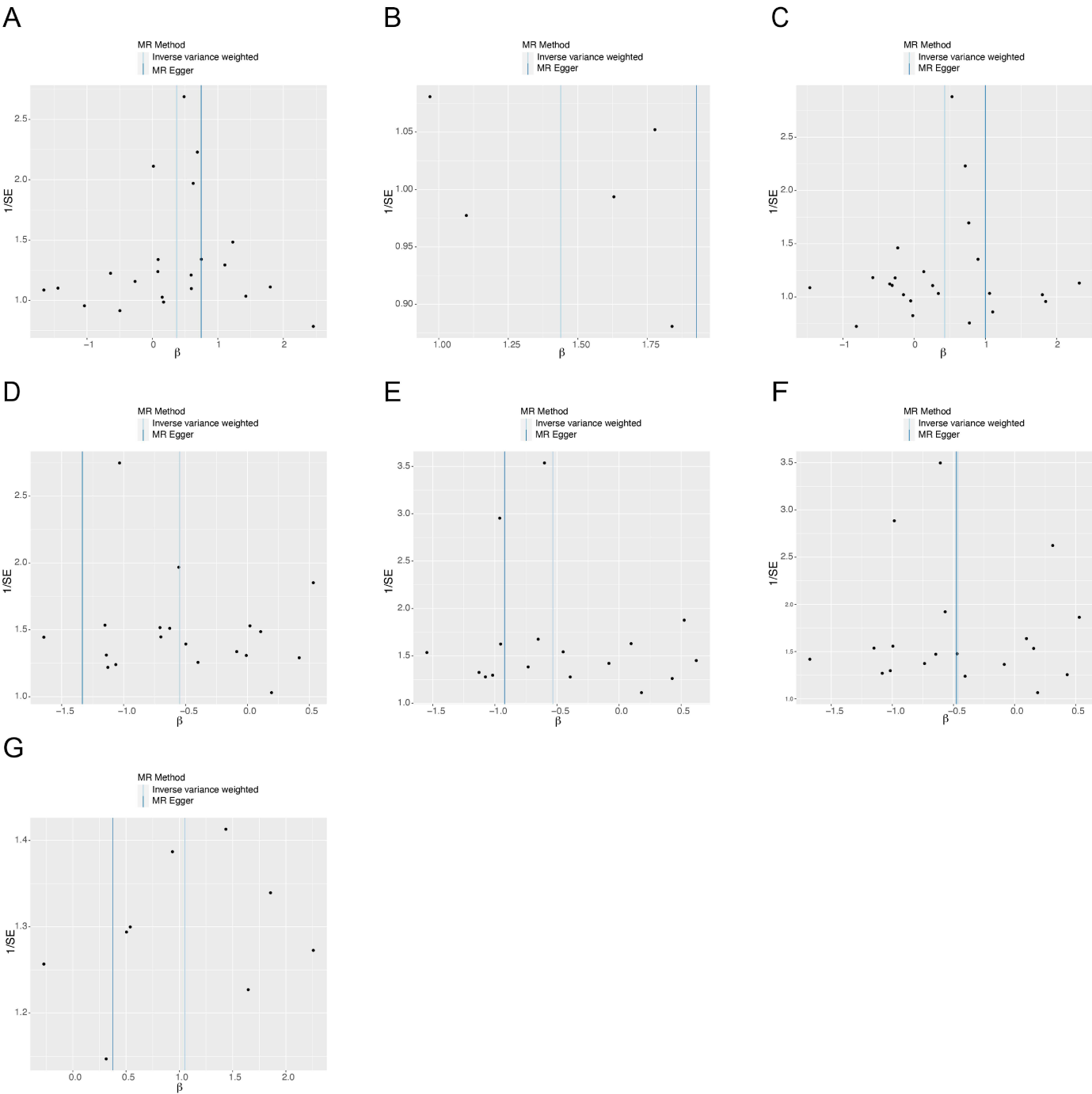

Supplement: Supplementary data [file wjps-2023-000754supp009.pdf]

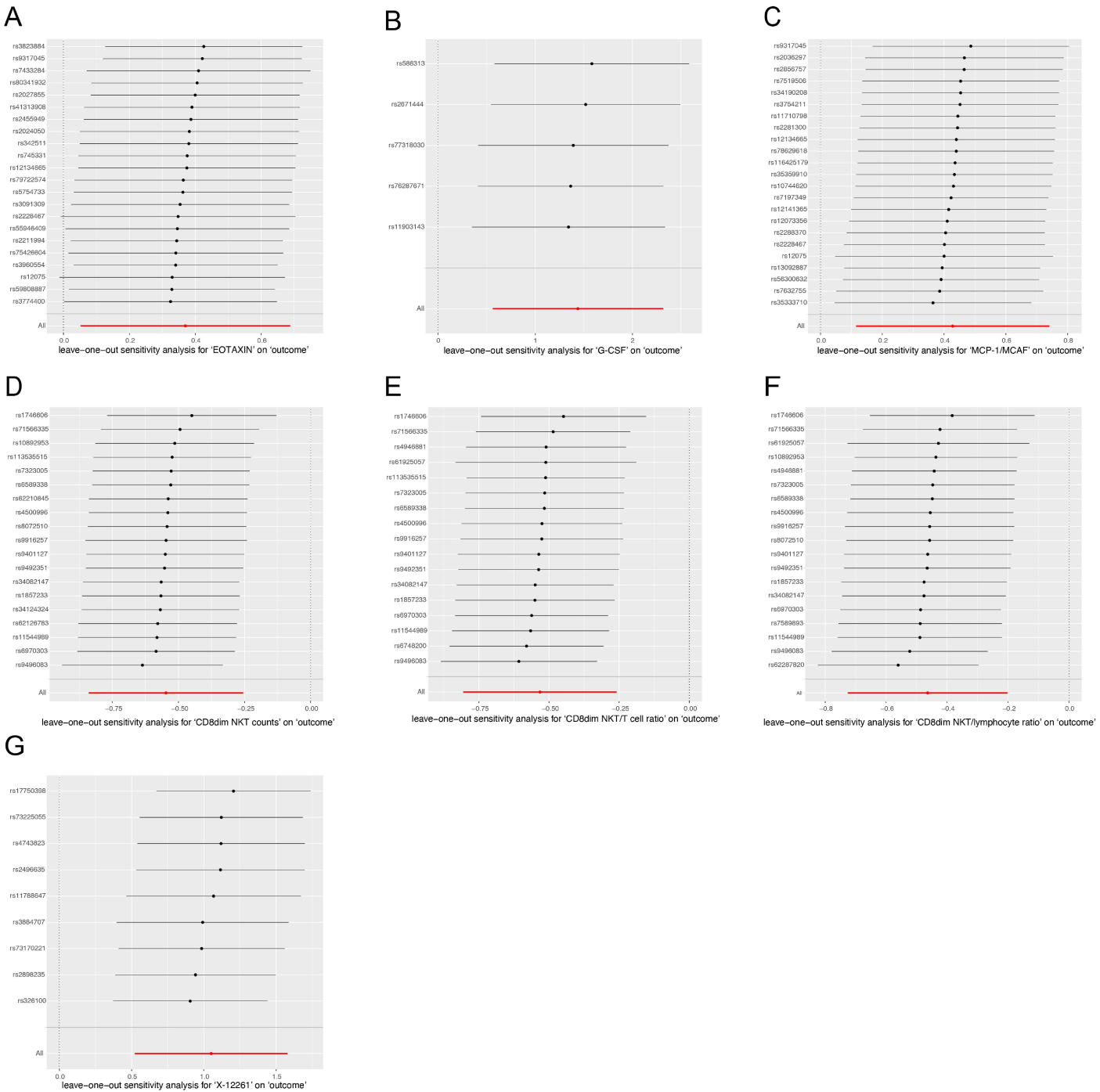

Supplement: Supplementary data [file wjps-2023-000754supp010.pdf]
